# Dr. Benjamin Dudley Lay

**Published:** 1868

**Authors:** 


### DR. BENJAMIN DUDLEY LAY.

This talented and excellent gentleman has, as we see from the proceedings of the Paducah Ky. Medical Society, removed from that city to locate permantly in Mobile, Ala. We commend him to the brotherhood of Medicine, as an educated physician—a man of letters—an honorable competitor, and a warm, unflinching friend, when friends are needed. He served the Confederacy with distinction from the beginning to the end of the war, and was one of the many accomplished Surgeons and Physicians who nobly sacrificed all else for the cause he loved.

